# Use of Electronic Entertainment and Communication Devices Among a Saudi Pediatric Population: Cross-Sectional Study

**DOI:** 10.2196/ijmr.9103

**Published:** 2018-09-06

**Authors:** Sami Omar Amawi, Ahmed Hussein Subki, Hazim Abdulkarim Khatib, Omer Sameer Alkhateeb, Rida Hashim Fida, Omar Mohammed Saggaf, Mohammed Mohammed Jan

**Affiliations:** ^1^ Department of Pediatrics College of Medicine King Abdulaziz University Jeddah Saudi Arabia

**Keywords:** television, tablet, mobile phone, computers, devices, entertainment, use, Saudi Arabia, children, population, behavior, parental impression

## Abstract

**Background:**

Excessive use of various electronic entertainment and communication devices, particularly among children, has been associated with increased behavioral problems. Despite children’s escalating use of these devices, parents’ awareness about the impact thereof is still lacking.

**Objective:**

The objective of this study was to assess the use of electronic entertainment and communication devices among children attending a health care facility in Jeddah, Saudi Arabia, as well as the parental impression regarding the impact of electronic devices use on the behavior of their child.

**Methods:**

A focused 15-item questionnaire was designed for this cross-sectional study involving mothers of children attending the Well Baby Clinic of King Abdulaziz University Hospital, Jeddah, Saudi Arabia from July 1, 2016 to November 30, 2016.

**Results:**

This study included 190 mothers. The mean ages of the children, mothers, and fathers were 7.3 (SD 3.5), 35 (SD 6.5), and 43 (SD 8.3) years, respectively. Most children were of Saudi Arabian nationality (106/190, 55.8%). The most used device in this study was television (154/190, 81.0%), followed by mobile phones (134/190, 70.5%), and tablets (116/190, 61.0%). Computers were the least used device in this study (59/190, 31.0%). In total, 24.7% (47/190) of children in this study used electronic entertainment and communication devices for more than 4 hours per day. Most mothers (129/190, 67.9%) felt that their child spends too much time on electronic devices. Hyperactivity or behavioral problems were reported by 20.0% (38/190) of mothers in this study. Children spending longer hours on electronic devices were much more likely to be perceived to suffer from hyperactivity or behavioral problems (*P*=.01).

**Conclusions:**

Parental awareness is necessary to counteract the harmful effects of using electronic devices for a prolonged period. Parents require help to cope with this problem effectively.

## Introduction

The continuous process of digitization of the world has led to an escalating use of various electronic entertainment and communication devices, including mobile phones, tablets, computers, and television (TV) [1,2]. A study in 2015 found that children who spent ≥2 hours daily on electronic devices were more likely to demonstrate an increased body mass index (BMI) [2]. A 2013 study compared the use of mobile phones among children in 4 different Arab countries and determined that around 80% of children aged between 8 and 18 years used mobile phones in Saudi Arabia. The study also ranked Saudi Arabia second after Egypt (87%) in high proportion of mobile phone use among children [3]. However, there is a scarcity of data about the use of electronic entertainment and communication devices among children and parental impression of behavior in Saudi Arabia.

The impact of screen addiction on health has been the subject of many studies. The results of these studies showed that excessive use of electronic devices, especially mobile phones and tablets had a harmful impact not only on the eyesight but also on the psycho-affective and cognitive development of the child. Moreover, the associated inactivity could trigger the development of some diseases, such as obesity and hypertension, at early ages [4-6]. TV viewing is the most widely studied behavior across the globe. This habit is, to some extent, correlated with childhood obesity [7,8]. A study conducted among 197 school children with a mean age of 8.7 (SD 2.1) years in the United Arab Emirates found that TV viewing and video game use for >2 hours per day was associated with behavioral problems such as being withdrawn, inattentive, and having externalizing behaviors, including hyperactivity and aggressive behaviors [9]. Higher aggression levels have also been observed in children watching TV programs or playing video games, especially with violent content [9]. On the contrary, an intervention study on elementary school students showed a reduction in aggressive behavior on watching TV for lesser hours [9]. Besides, excessive use of electronic devices could adversely affect a child’s sleep-wake cycle, resulting in further behavioral problems and occasionally stereotypical motor behaviors [1,10,11]. This is particularly important given the extent of use of such devices in our community and their potential negative impacts on children’s well-being. Parental awareness about the harmful effects of electronic and communication devices on children is tremendously important for preventing their occurrence [12,13].

The objective of this study was to assess the level of use of electronic devices among children attending a health care facility in Saudi Arabia and the parents’ perspectives about the impact of screen use on the behavior of their children.

## Methods

A cross-sectional survey involving mothers of children attending the Well Baby Clinics of King Abdulaziz University Hospital (KAUH), Jeddah, Saudi Arabia was conducted from the 2016 July 1 to 2016 November 30. KAUH is the main university teaching hospital of the Western Region of Saudi Arabia. It is a large multi-specialty adult and pediatric (children aged 8-15 years) hospital that also provides primary medical care and immunization for all children born at the hospital. Three Well Baby Clinics per week provide parental support, education, and routine immunizations.

For this survey, 190 participants were chosen by convenience sampling and all 190 mothers responded to the questionnaires, thus providing a response rate of 100%. Inclusion criteria were as follows: mothers with one or more child who used electronic entertainment devices and was willing to participate in this survey. No exclusion criteria were set.

Because we could not find any validated questionnaires about the impact of electronic devices use on children behavior, we developed a focused 15-item questionnaire to examine the use of various electronic entertainment and communication devices. The questionnaire also assessed parental supervision and interaction. The questionnaire was not validated. The first part of the questionnaire collected sociodemographic characteristics such as the age of the child and the parents, the parents’ education, occupation, family income, and nationality. The second part was relevant to the type of electronic devices used by the child, the time spent by children on those devices, and whether the parents noticed any change in the behavior of their children while using these devices (see Multimedia Appendix 1).

The study and questionnaire were explained to the mothers before they voluntarily participated in the study. The identity of the participants was not requested to ensure privacy and encourage accurate responses. The bioethical committee of KAUH approved the study design and questionnaire.

Participants filled a paper-pencil survey; later, we entered the data into an Excel sheet. Statistical analyses were performed using the Statistical Package for Social Sciences version 20 (SPSS Inc, Chicago, IL, USA). Frequencies and percentages were used for the descriptive analyses. Association between categorical variables was examined using chi-square test. Statistical significance was defined as *P* values of <.05.

## Results

This study included 190 mothers. The mean ages of the children, mothers, and fathers were 7.3 (SD 3.5), 35 (SD 6.5), and 43 (SD 8.3) years, respectively. Most children were of Saudi Arabian nationality (106/190, 55.8%). Most mothers were housewives (145/190, 76.3%), and 40.5% (77/190) of the mothers in our study had school-level education. Most fathers were employed (135/190, 71.0%), and 53.1% (101/190) of the fathers in our study had a university-level education. Of the families in the study, 42.1% (80/190) had a monthly income of <5000 SAR (<US $1300; Table 1).

The most used device in this study was TV (154/190, 81.0%), followed by mobile phones (134/190, 70.5%) and tablets (116/190, 61.0%). Computers (59/190, 31.0%) were the least used device in this study. In total, 24.7% (47/190) of children in this study used electronic entertainment and communication devices for than 4 hours (Table 2).

When asked if the parents would limit the time spent on these devices, about 63.1% (120/190) answered “yes” (16% responded “sometimes” and 47% responded “always”). Most mothers (129/190, 67.9%) felt that their child spends too much time on electronic devices. Hyperactivity or behavioral problems were reported by 20.0% (38/190) of mothers in this study. Overall, 10.0% (19/190) of the mothers “sometimes” observed hyperactivity or behavioral problems in their children; similarly, 10.0% (19/190) of the mothers “always” observed hyperactivity or behavioral problems in their children (Table 3).

**Table 1:** Sociodemographic characteristics of the study families (N=190).

Characteristics		Value
**Age (years), mean (SD), range**		
	Child	7.3 (3.5), 1-16
	Mother	35 (6.5), 24-50
	Father	43 (8.3), 28-63
**Nationality of child, n (%)**		
	Saudi Arabia	106 (55.8)
	Yemen	19 (10.0)
	Sudan	17 (8.9)
	Other nationalities	48 (25.3)
**Mother’s employment, n (%)**		
	Employed	45 (23.7)
	Unemployed	145 (76.3)
**Father’s employment, n (%)**		
	Employed	135 (71.0)
	Unemployed	55 (28.9)
**Mother’s educational level, n (%)**		
	None	30 (15.8)
	School grade	83 (43.7)
	College or university	77 (40.5)
**Father’s educational level, n (%)**		
	None	29 (15.3)
	School grade	60 (31.6)
	College or university	101 (53.1)
**Monthly income of family, n (%)**		
	<5000 SAR (<US $1333)	80 (42.1)
	5000-10,000 SAR (US $1333-2666)	57 (30.0)
	10,000 SAR (>US $2666)	53 (27.9)

**Table 2:** Most frequently used devices and overall time spent on electronic entertainment and communication devices (N=190).

Most used device		n (%)
**Television**		
	Yes	154 (81.0)
	No	36 (18.9)
**Mobile phones**		
	Yes	134 (70.5)
	No	56 (29.5)
**Tablets**		
	Yes	116 (61.0)
	No	74 (38.9)
**Computers/laptops**		
	Yes	59 (31.0)
	No	131 (68.9)
**Overall time spent on electronic entertainment and communication devices**		
	1 hr per day	61 (32.1)
	2-4 hr per day	82 (43.1)
	4-6 hr per day	25 (13.1)
	>6 hr per day	22 (11.6)

**Table 3:** Parent-child interaction and behavioral impression (N=190).

Behavioral impression and parent-child interaction		n (%)
**Do you try to limit the time your child spends on these devices?**		
	Never	70 (36.8)
	Sometimes	30 (15.8)
	Always	90 (47.4)
**Do you think your child spends too much time on these devices?**		
	Yes	129 (67.9)
	No	61 (32.1)
**Does your child suffer from hyperactivity or behavior problems?**		
	Yes	38 (20.0)
	No	152 (80.0)
**How often do you observe hyperactivity or behavior problems problem in your child?**		
	Never	152 (80.0)
	Sometimes	19 (10.0)
	Always	19 (10.0)

Time spent on electronic devices correlated significantly with parental impression that their child was suffering from behavioral problems. In total, 8.4% (16/190) of mothers in our study whose children spent >2 hours per day on electronic devices reported that their children “always” exhibited behavioral problems. On the other hand, only 1.6% (3/190) mothers whose children spent 1 hour per day on electronic devices reported that their children “always” had behavioral problems. Children who spent longer hours on electronic devices were much more likely to be perceived to suffer from hyperactivity or behavioral problems (*P*=.01; Table 4).

**Table 4:** Association between time spent on electronic devices and parental impression that the child suffered from hyperactivity or behavioral problems (N=190; *P*=.01).

Time spent on electronic devices (hr per day)	Hyperactivity or behavioral problems, n (%)		
	None (n=152)	Sometimes (n=19)	Always (n=19)
1 (n=61)	54 (88)	4 (7)	3 (5)
2-4 (n=82)	67 (82)	8 (10)	7 (8)
4-6 (n=25)	16 (64)	6 (24)	3 (12)
>6 (n=22)	15 (68)	1 (5)	6 (27)

## Discussion

### Principal Findings

Our study confirmed that many families living in Saudi Arabia were affected by their children’s excessive use of various electronic entertainment and communication devices. When asked if the parents would limit the time spent on these devices, about 63.1% (120/190) answered “yes” and mothers (129/190, 67.9%) felt that their child spends too much time on electronic devices. In fact, in 24.7% (47/190) of children, the time spent on electronic devices was >4 hours per day. After TV viewing, mobile phones were the second most frequently used device. Previous research identified that less time spent on various electronic and communication devices resulted in prevention of depression in young adults [14], thereby potentially improving health during adulthood. In addition, other investigators found correlations with several future neurological disorders, such as recurrent headaches and migraine [15].

One out of 5 mothers (38/190, 20.0%) thought their child was experiencing hyperactivity or behavioral problems. This was queried in a direct manner and not examined in a structured way or through the use of a standardized diagnostic inventory. It was simply based on the mother’s overall impression of her child’s behavior. The accuracy of such global impressions has been proven to be effective in other studies [16]. Watching TV, which was most prevalent (154/190, 81.0%) in our sample, was previously noted by other investigators who found no association between watching TV and emotional symptoms, hyperactivity, inattention, or peer relationship problems [17]. However, previous research found an association between watching TV for >2 hours per day and antisocial behaviors, aggressive personalities, and criminal convictions [18]. This might be explained by cultural differences or inclusion of older children and adolescents in those studies.

### Limitations

This study had several limitations that warrant discussions. Firstly, our sample size was relatively small. A larger study could provide more insight about potential correlations with sociodemographic variables such as parents’ ages, educational levels, family incomes, and nationalities. Secondly, because we used convenience sampling from one region in Saudi Arabia (Jeddah), our findings might not be representative of other regions of Saudi Arabia. Moreover, the study participants comprised working-class people with average educational and economic levels. This, in turn, limited our ability to generalize our findings to the population at large. Hence, more detailed research, involving different regions of the country and different groups of people according to educational qualifications, income levels, and origins, should be conducted to attain a generalizable conclusion. Thirdly, our questionnaire was not used or validated previously, mainly owing to the limited availability of relevant literature. The questionnaire did not include specific behavioral problems that were observed. Therefore, the use of structured behavioral inventories is needed to confirm the link between hyperactivity or behavioral disorders and excessive use of different electronic entertainment and communication devices. Another possible limitation of this study, like most other cross-sectional studies, was self-reporting. This rendered the information obtained regarding the time spent by the children on electronic devices questionable [2]. Additionally, the information was collected from the mothers only, rather than the children themselves and there might be a probability that the mothers exaggerated their responses. Even though several previous population-based studies utilized mother-reported screen time, there are concerns regarding the validity and reliability of these reports [17]. Furthermore, this study did not assess the content of the electronic and communication devices. Earlier studies revealed that aggressive content might be responsible for psychological or behavioral problems in children, regardless of how much time was spent watching TV [6]. However, we did not assess whether the screen contents were age-appropriate or whether they negatively impacted the children’s behaviors. Therefore, it is essential to survey the contents of the electronic and communication devices to refine the results of the study. Despite these limitations, the major strength of this study was that this was the first study that attempted to survey children in Jeddah, Saudi Arabia to determine how much time they spent on electronic entertainment and communication devices.

### Conclusions

In conclusion, this study detected excessive use (2-6 hours or more per day) of electronic devices by the children in the study sample. The time spent is higher than the recommended upper limit of 2 hours per day according to American Academy of Pediatrics (AAP). In fact, using communication devices >2 hours per day is linked with significant behavioral problems [9]. Accordingly, the parents in this study reported increased behavioral problems or hyperactivity among children who spent more time on electronic and communication devices. Our results indicate that the parents should follow the recommendations of AAP to limit their children’s exposure to electronic devices to no more than >2 hours per day, potentially reducing the risk of developing behavioral abnormalities [19]. Through increased parental awareness, the possible detrimental effects of technology on children’s psychological development and behaviors could be counteracted [9]. This in turn might help to reduce the overall time spent on electronic devices [6]. Nonetheless, to deal with this issue efficiently, parents require help. Public educational campaigns are imperative for addressing this growing problem and providing effective solutions. Finally, slower-paced, nonviolent educational programs are believed to have less negative impact on children’s behaviors [19]. Therefore, besides monitoring screen time, parents should monitor screen content. Further studies must be attempted to detect the influence of the screen content on children’s behavioral patterns.

## Abbreviations

AAP: American Academy of Pediatrics
BMI: body mass index
KAUH: King Abdulaziz University Hospital
TV: television

## Appendix

Multimedia Appendix 1 Questionnaire.
