# Supplementary material for: Use of Electronic Entertainment and Communication Devices Among a Saudi Pediatric Population: Cross-Sectional Study
Source: Interact J Med Res. 2018 Sep 6;7(2):e13. doi: 10.2196/ijmr.9103 (PMC6242707; doi:10.2196/ijmr.9103)
Supplement: Multimedia Appendix 1 [file ijmr_v7i2e13_app1.pdf]

Multimedia Appendix 1

The following is a short questionnaire to be filled out, if you approve to participate in this study. Our research aims to survey the extent of the use of electronic entertainment and communication devices amongst children in Saudi Arabia.

Do you agree to participate in this study? *Yes * No

<u>If yes, please answer the following questions:</u>

What is your child's age? ……

Mother's age …… Mother's education: *None * School * College\University

Father's age …… Father's education: *None * School * College\University

Family's Monthly income?

*<5000 SAR (<1333 USD)

*5000-10,000 SAR (1333-2666 USD)

*> 10,000 SAR (> 2666 USD)

Child's nationality: …………

Please circle the electronic entertainment and or communication device that your uses?

*Television *Cellphones *Electronic tablets *Computers

How long does your child spend daily using these devices?

*1 hour *2-4 hours *4-6 hours *more than 6 hours

Does your child suffer from hyperactivity or behavior problems? *Yes *No

How often do you observe hyperactivity or behavior problems problem in your child? * Never * Sometimes *Always

Do you try to limit the time your child spends on these devices? Never * Sometimes *Always

Do you think that your child spends too much time on these devices? *Yes *No

Thank you for participating in our study
